# Is Night Shift Work Associated with Ovarian Cancer? A Systematic Review and Meta-Analysis

**DOI:** 10.3390/medsci13040228

**Published:** 2025-10-12

**Authors:** Ahmed Arafa, Mazin Alhussein, Amin Alayyan, Haytham A. Sheerah, Mona S. Ibrahim, Abeer S. Alasmari, Sarah A. Barzanji, Samah A. Bukhari, Alhanouf K. Althaydi, Ehab Elkady, Tarig A. Y. Ali, Abdulrahman Almazrooa

**Affiliations:** 1 Department of Preventive Cardiology, National Cerebral and Cardiovascular Center, Suita 564-8565, Osaka, Japan; 2 Department of Public Health and Community Medicine, Faculty of Medicine, Beni-Suef University, Beni-Suef 62521, Egypt; 3 Nottingham University Hospitals NHS Trust, Nottingham NG5 1PB, UK; 4 University Hospitals of Leicester NHS Trust, Leicester LE5 4PW, UK; 5 Chief Executive Office, Public Health Authority, Riyadh 11451, Saudi Arabia; hasheerah@moh.gov.sa; 6 Population Health Deputyship, Ministry of Health, Riyadh 11451, Saudi Arabia; 7 Department of Obstetrics and Gynecology, Dhurma Hospital, Riyadh 11211, Saudi Arabia; monasi@moh.gov.sa; 8 National Breastfeeding Program, Ministry of Health, Riyadh 11451, Saudi Arabia; 9 King Abdulaziz Medical City, Jeddah 22384, Saudi Arabia; alasmariabs@mngha.med.sa (A.S.A.); barzanjisa@mngha.med.sa (S.A.B.); sbukhari@bu.edu (S.A.B.); 10 Department of Obstetrics and Gynecology, Security Forces Hospital, Riyadh 11481, Saudi Arabia; aalthaydi@moi.med.sa; 11 Ada’a Health Center, Ministry of Health, Riyadh 11451, Saudi Arabia; eelkady@moh.gov.sa (E.E.); tabali@moh.gov.sa (T.A.Y.A.); 12 Department of Anesthesia and Critical Care, Faculty of Medicine, King Abdulaziz University, Jeddah 21589, Saudi Arabia; aaalmazrooa@kau.edu.sa

**Keywords:** night shift, cancer prevention, ovarian cancer, systematic review, meta-analysis, occupational risk, epidemiological evidence

## Abstract

**Background**: Night shift work has been classified as a probable carcinogen due to its disruption of circadian rhythms. However, whether night shift work can increase the risk of ovarian cancer remains unclear. Herein, we investigated this association using a systematic review and meta-analysis. **Methods**: We systematically searched several databases until June 2025 for relevant studies. Effect estimates were extracted and pooled using a random-effects model to calculate odds ratios (ORs) with 95% confidence intervals (CIs). Heterogeneity across studies was assessed using the *I*^2^ statistic, and publication bias was assessed using Egger’s regression test and funnel plot asymmetry. **Results**: Seven studies (eight cohorts) involving >2.5 million women were included. Overall, night shift work was not significantly associated with ovarian cancer (OR = 1.13; 95% CI: 0.96, 1.32; *I*^2^ = 49%). However, significant associations were observed in case–control studies (OR = 1.36; 95% CI: 1.12, 1.66; *I*^2^ = 0.8%) and in high-quality studies (OR = 1.17; 95% CI: 1.00, 1.37; *I*^2^ = 52%). Sensitivity analyses suggested that exposure misclassification in some cohort studies attenuated risk estimates. No publication bias was detected (z = −0.63, *p* = 0.53). **Conclusions**: While the overall findings did not demonstrate a statistically significant association, evidence from case–control studies that collected detailed information about night shift work suggests an increased ovarian cancer risk in night shift workers. Future large-scale prospective studies with detailed exposure assessments are warranted to confirm these findings.

## 1. Introduction

Ovarian cancer ranks as the eighth most common cancer among women worldwide, with an estimated 324,398 new cases and 206,839 deaths reported in 2022 [1]. Unlike breast or cervical cancer, where screening programs and early detection strategies exist, ovarian cancer is often diagnosed at an advanced stage, resulting in poor survival outcomes. This late detection contributes not only to high mortality, but also to substantial healthcare costs and productivity losses, with treatment requiring extensive surgery, chemotherapy, and prolonged hospitalizations. Identifying modifiable risk factors is therefore a priority in efforts to reduce the global burden of ovarian cancer [1,2].

While established risk factors for ovarian cancer include advancing age, a family history of the disease, *BRCA1/2* mutations, reproductive factors, and hormone use, there is growing interest in the role of occupational exposures in ovarian carcinogenesis [3]. Among these, night shift work has garnered attention due to its potential to disrupt circadian rhythms, which may, in turn, influence cancer development. The International Agency for Research on Cancer (IARC) has classified night shift work as a probable human carcinogen (Group 2A), citing evidence for its role in disturbing endogenous circadian regulation [4]. Epidemiological studies have linked night shift work to increased risks of several cancers, including breast [5], prostate [6], esophageal [7], and colorectal cancers [8].

Mechanistically, the suppression of melatonin, a hormone with antioxidant and oncostatic properties, caused by exposure to light at night, is the main biological rationale behind these associations. It may hinder DNA repair, increase oxidative stress, and alter estrogen signaling, all of which are associated with hormone-sensitive cancers [9]. Given the presence of estrogen receptors on ovarian tissue and evidence of circadian gene dysregulation in ovarian tumor cells, there is biological plausibility for a link between circadian disruption and ovarian carcinogenesis [10].

Despite these potential mechanisms, epidemiological evidence on the association between night shift work and ovarian cancer remains inconsistent. Some studies have reported increased risks among women exposed to night shift work [11,12], while others have observed no association [13,14,15,16,17]. Additionally, a previous meta-analysis investigating the association between shift work and several cancers, including ovarian cancer, did not reach conclusive findings [18]. In this context, we conducted an updated systematic review and meta-analysis of observational studies to evaluate the association between night shift work and the risk of ovarian cancer.

## 2. Materials and Methods

### 2.1. Registration

This systematic review and meta-analysis adhered to the Preferred Reporting Items for Systematic Reviews and Meta-Analyses (PRISMA) guidelines [19]. The protocol was registered in the International Prospective Register of Systematic Reviews (PROSPERO) with the ID CRD420241066955.

### 2.2. Eligibility Criteria

We included observational studies that met the following criteria: (1) the exposure was night shift work, including night, rotating, or irregular work schedules; (2) the outcome was incident or fatal ovarian cancer; (3) the study design was cohort or case–control; (4) effect estimates such as odds ratios (ORs), relative risks, or hazard ratios with 95% confidence intervals (CIs) were reported; and (5) articles were published in English. We excluded reviews, case reports, editorials, animal studies, and duplicate publications.

### 2.3. Search Strategy

A systematic search was conducted on PubMed, Web of Science, and Scopus for articles published before 5 June 2025, using predefined search terms related to night shift work and cancer (Table A1). Two reviewers independently screened titles and abstracts for eligibility. Full texts of relevant articles were reviewed, and reference lists of included articles and relevant reviews were checked for additional studies. Full texts of potentially relevant studies were then assessed against the inclusion criteria. Disagreements were resolved through discussion.

### 2.4. Data Extraction

Two reviewers independently extracted data using a pre-designed form. Variables included the following: first author, publication year, country, study design, follow-up period, sample size, population details, definitions and assessment methods for night shift work, outcome definitions, adjusted covariates, and effect estimates with 95% CIs. When multiple estimates were available, the most fully adjusted was used.

### 2.5. Quality Assessment

Study quality was assessed using a modified version of the Newcastle–Ottawa Scale (NOS), which scores on a 9-star system [20]. The scale covers selection, comparability, and outcome/exposure domains, with a maximum score of 9. Scores of 7–9 indicated high quality, 4–6 moderate quality, and 0–3 low quality. Independent assessments were conducted by two authors, with disagreements resolved through consensus.

### 2.6. Statistical Analysis

The most adjusted risk estimates for the highest dose or frequency of night shift work from each study were pooled using a random-effects model (DerSimonian and Laird method) [21]. Heterogeneity was evaluated using τ^2^, *I*^2^, and H^2^ statistics [22]. Publication bias was assessed with Egger’s regression test and funnel plot asymmetry [23]. Sensitivity analyses, excluding one study at a time, tested the robustness of the results. The subgroup analyses examined study design (cohort vs. case–control), exposure assessment method (questionnaire vs. job-exposure matrix), study quality, and outcome type (incidence vs. mortality). All analyses were performed using R software (version 3.2.0) with the ‘metafor’ package [24].

## 3. Results

### 3.1. Study Selection

**Figure 1:**
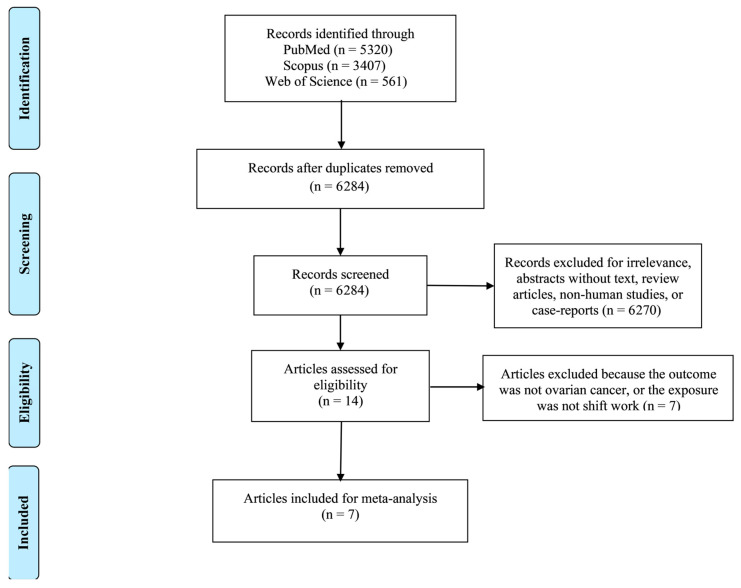
PRISMA flow diagram.

After removing duplicates, reviews, and studies with unrelated exposures or outcomes, seven studies (comprising eight cohorts) were included in the meta-analysis (Figure 1).

### 3.2. Study Characteristics

The included studies were published between 2007 and 2020 and were conducted in North America and Northern Europe (the US, Canada, Denmark, and Sweden). Five studies had a cohort design [12,13,14,16,17], while two were population-based case–control studies [11,15]. Across all studies, 2,533,187 women were included. Night shift work was assessed either through self-administered questionnaires or job-exposure matrices, and the outcomes included both ovarian cancer incidence and mortality. Most studies adjusted for relevant confounders such as age, parity, oral contraceptive use, and body mass index (Table 1). Of the eight cohorts, two studies (Bhatti et al. [11] and Carter et al. [12]) reported a statistically significant positive association between night shift work and ovarian cancer. The remaining six cohorts found no significant association [13,14,15,16,17]. Using the modified NOS, all included studies were rated as having moderate or high quality (Table 2).

### 3.3. Results of Syntheses

**Figure 2:**
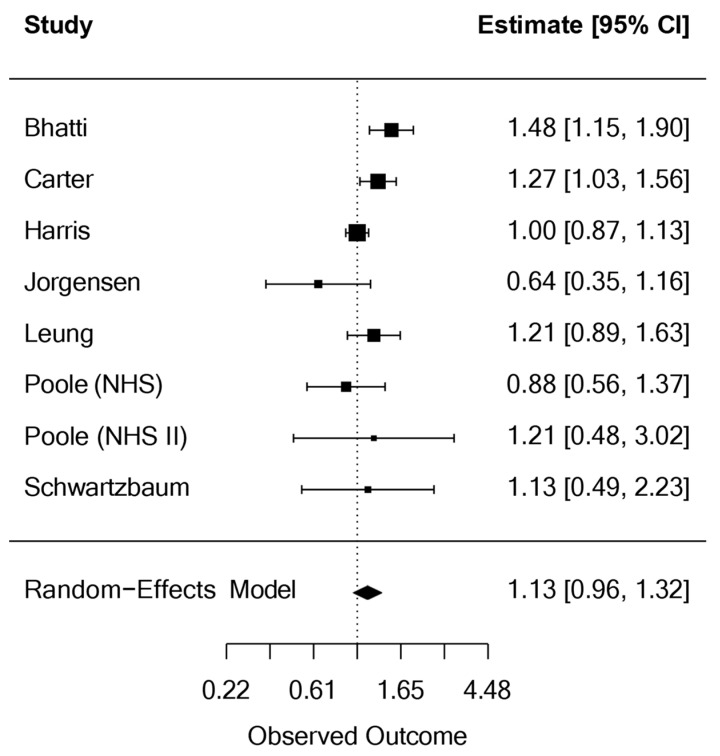
Meta-analysis of the association between night shift work and ovarian cancer.

The contribution of each cohort to the overall meta-analysis weight was as follows: Bhatti et al. [11]: 17.6%; Carter et al. [12]: 20.4%; Harris et al. [13]: 25.8%; Jørgensen et al. [14]: 5.8%; Leung et al. [15]: 14.7%; Poole et al. (NHS) [16]: 9.0%; Poole et al. NHS II [16]: 2.8%; and Schwartzbaum et al. [17]: 3.9%. The pooled analysis of all seven studies (eight cohorts) found no statistically significant association between night shift work and ovarian cancer: pooled OR = 1.13 (95% CI: 0.96, 1.32). Moderate but statistically non-significant heterogeneity was observed across studies (τ^2^ = 0.02, *I*^2^ = 49.04%, H^2^ = 1.96; *p* = 0.056) (Figure 2).

### 3.4. Publication Bias

**Figure 3:**
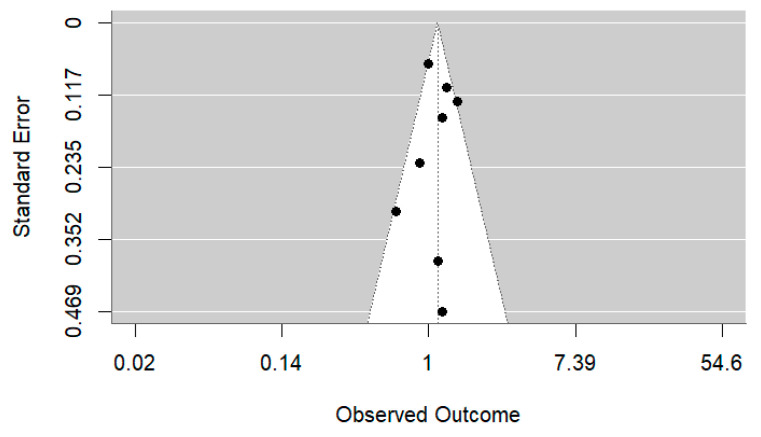
Funnel plot of the studies investigating the association between night shift work and ovarian cancer.

Visual inspection of the funnel plot (Figure 3) and results from Egger’s regression test indicated no evidence of publication bias (z = −0.626, *p* = 0.531), suggesting that small-study effects were unlikely to have influenced the overall results.

### 3.5. Sensitivity Analyses

We conducted leave-one-out analyses to assess the influence of individual studies. The exclusion of Jørgensen et al. [14] made the overall estimate statistically significant: (OR = 1.16; 95% CI: 1.00, 1.35). Removing Bhatti et al. [11] substantially reduced heterogeneity (*I*^2^ = 24.11%), suggesting that it was a key source of variability (Table 3).

### 3.6. Subgroup Analyses

Stratification by study design indicated a positive association in case–control studies (OR = 1.36; 95% CI: 1.12, 1.66; *I*^2^ = 0.83%), but not in cohort studies (OR = 1.04; 95% CI: 0.89, 1.23; *I*^2^ = 29.62%). Confining the analysis to high-quality studies, as indicated by modified NOS, made the association statistically significant (OR = 1.17; 95% CI: 1.00, 1.37; *I*^2^ = 52.01%) (Table 4).

## 4. Discussion

This systematic review and meta-analysis synthesizes data from over 2.5 million women to evaluate the association between night shift work and ovarian cancer. Although the overall pooled estimate did not reach statistical significance, subgroup analyses revealed important patterns. Case–control studies and high-quality studies showed a statistically significant positive association, suggesting that methodological differences may account for the inconsistencies observed across the literature.

The stronger associations observed in case–control studies may reflect more detailed and accurate exposure assessment. These studies typically collected retrospective information on the frequency and duration of night shift work, thereby reducing exposure misclassification. In contrast, cohort studies assessed night shift work only at baseline, relying on current job titles or occupational codes without accounting for cumulative exposure or changes over time. These methodological limitations likely introduced non-differential misclassification, biasing results toward the null.

Still, recall bias is a notable concern in retrospective designs, as cancer patients may recall or report their occupational history differently than healthy controls, potentially inflating associations. Such bias can arise from differential memory, selective reporting, or increased awareness of suspected risk factors after diagnosis [25,26]. Evidence from breast cancer research shows that women who believed that shift work increased cancer risk were more likely to report past exposure to shift work, suggesting that personal beliefs may influence exposure reporting. However, the study also shows that prompting participants to recall shift work did not increase the likelihood of believing in its carcinogenic potential, indicating that the observed association between belief and reporting was more likely due to exposed individuals endorsing the exposure–disease link rather than classical recall bias [27].

Moreover, study quality also appeared to influence results. High-quality studies (as rated by the modified NOS) showed a significant association, while moderate-quality studies did not. This suggests that rigorous exposure assessment, appropriate control selection, and comprehensive adjustment for confounders may be critical in detecting true associations between night shift work and ovarian cancer.

Several mechanisms support a potential link between night shift work and ovarian carcinogenesis. Disruption of the circadian rhythm suppresses nocturnal melatonin production, a hormone with known oncostatic properties, including antioxidant activity, inhibition of cell proliferation, and modulation of estrogen receptor expression [9,28]. Reduced melatonin levels can lead to hyperestrogenism, a recognized risk factor for hormone-sensitive malignancies such as ovarian cancer [28]. Additionally, chronic exposure to light at night may elevate systemic inflammation and oxidative stress, both of which are implicated in tumorigenesis [29]. Altered sleep–wake cycles can also result in metabolic dysfunction, including insulin resistance and abnormal adipokine signaling, further contributing to an environment conducive to malignant transformation [30]. Furthermore, circadian misalignment can dysregulate the expression of core clock genes such as *CLOCK*, *BMAL1*, *PER*, and *CRY*, which play vital roles in cell cycle regulation, DNA damage repair, and apoptosis. Dysregulation of these genes has been observed in ovarian tumor tissues and is thought to promote carcinogenesis by impairing genomic stability and enhancing proliferation [31].

From a public health perspective, while this analysis does not conclusively establish night shift work as a risk factor for ovarian cancer, the positive associations observed in specific study subgroups raise important concerns. Millions of women worldwide are engaged in night shift work, particularly in the healthcare and service industries. Even a modest increase in cancer risk could have significant population-level implications. Although current screening guidelines for ovarian cancer do not consider occupational history [32], clinicians should consider night shift work when assessing overall risk, particularly for women with additional risk factors such as family history or *BRCA* mutations.

It is also important to consider health equity. Women in lower socioeconomic groups and minority populations are disproportionately represented in night shift work occupations, which may exacerbate existing disparities in cancer outcomes [33]. This environmental justice perspective strengthens the rationale for workplace interventions and targeted preventive strategies.

The main strength of this study is the large-pooled sample, which enabled precise effect estimates and subgroup analyses. However, some limitations should be addressed. First, there was heterogeneity in study design, populations, and how night shift work was defined and measured. Most studies lacked detailed exposure metrics that would allow for a proper assessment of the intensity, frequency, and duration of night shift work. Some studies relied on current job titles or general occupational classifications without lifetime work histories, resulting in potential misclassification. For example, Carter et al. [12] classified exposure based on the current job and had no data on prior night shift work or exposure frequency. Harris et al. [13] used proxy occupational categories rather than direct night shift work data. Jørgensen et al. [14] had no night shift work duration data and likely included misclassification due to early-career night shift work in older nurses. Leung et al. [15] reported a relatively small number of long-term exposed cases and potential selection bias due to low control participation, which may have skewed the exposure prevalence. Poole et al. [16] potentially misclassified permanent night workers as unexposed, biasing results toward the null. Schwartzbaum et al. [17] used aggregated job-level data instead of individual exposure histories. Second, few studies distinguished fixed-night work from rotating night shift work; therefore, we could not examine it separately, and our findings mainly reflect rotating shift schedules. Third, generalizability may be restricted, as the studies included were conducted on Western populations.

## 5. Conclusions

While our meta-analysis did not confirm a statistically significant association between night shift work and ovarian cancer overall, evidence from case–control and higher-quality studies suggested a possible increased risk. To confirm this association, future research should focus on large-scale prospective cohort studies with standardized and repeated assessments of night shift work exposure, detailed adjustment for established ovarian cancer risk factors, and integration of biological investigations to explore pathways linking circadian disruption to ovarian carcinogenesis.

## Figures and Tables

**Table 1:** Summary of the studies included in the meta-analysis.

Study ID	Study Design (Follow-Up) Outcome	Population	Night Shift Work Categories (Assessment Method)	Covariates
Bhatti et al. [11] (2013) US	Population-based case–control Cancer incidence	1490 cases and 1832 controls, aged 35–74 years	Night shift (ever) vs. never (questionnaire)	Age, county, reference year, parity, OC, and BMI
Carter et al. [12] (2014) US	Cohort (1982–2010) Cancer mortality	161,004 women from CPS-II with a mean age of 50.3 years	Rotating shift vs. fixed day (questionnaire)	Age, OC, age at menarche, age at menopause, tubal ligation, parity, HRT, race, BMI, PA, and family history of breast/ovarian cancer
Harris et al. [13] (2020) Canada	Cohort (1991–2010) Cancer incidence	939,520 women from CanCHEC, aged 25–74 years	Evening, rotating, or regular night shifts (>50% vs. 0–5%) (job-exposure matrix)	Age, provincial region, education, and parity
Jørgensen et al. [14] (2017) Denmark	Cohort (1993–2013) Cancer mortality	28,731 women from the DNC, aged ≥44 years	Rotating (day, evening, and night) vs. day shifts (questionnaire)	Age, smoking, PA, BMI, alcohol, diet, chronic diseases, health status, stressful work, marital status, parity, age at first birth, HRT, and OC
Leung et al. [15] (2019) Canada	Population-based case–control Cancer incidence	496 cases and 906 controls from PROVAQ, aged 18–79 years	Shift 12+ years vs. never	Age, education, and parity
Poole et al. [16] (2011) US	Cohort (NHS: 1988–2008 and NHS II: 1989–2007) Cancer incidence	181,548 women from NHS, aged 30–55 years, and 68,999 women from NHS II, aged 25–42 years	NHS: Rotating night shift 20+ years vs. none NHS II: Rotating night shift 15–19 years vs. none (questionnaire)	Age, OC, parity, BMI, smoking, tubal ligation, menopausal status, family history of ovarian cancer, county, and breastfeeding
Schwartzbaum et al. [17] (2007) Sweden	Cohort (1971–1989) Cancer incidence	1,148,661 women registered in the 1960 and the 1970 census data	Shift work (standardized incidence ratio) (job-exposure matrix)	Age, socioeconomic status, job, and residence

BMI: Body mass index; CanCHEC: Canadian Census Health and Environment Cohort; CPS-II: Cancer Prevention Study II; DNC: Danish Nurses Cohort; HRT: Hormone replacement therapy; OC: Oral contraceptives; PA: Physical activity; PROVAQ: PRevention of OVArian Cancer in Quebec study; NHS: Nurses’ Health Study.

**Table 2:** Quality assessment of the studies included in the meta-analysis using the Newcastle–Ottawa Scale.

Cohort Studies					
**Items**	**Carter et al.** [12]	**Harris et al.** [13]	**Jørgensen et al.** [14]	**Poole et al.** [16]	**Schwartzbaum et al.** [17]
Representativeness of the exposed cohort	*	*	*	*	*
Ascertainment of exposure	--	**--**	**--**	**--**	**--**
Selection of the non-exposed cohort	*	*	*	*	*
Demonstration that the outcome of interest was not present at the start of the study	*	*	*	*	*
Comparability	**	*	*	**	*
Assessment of outcome	*	*		*	**--**
Follow-up long enough for outcomes to occur	*	*	*	*	*
Adequacy of follow-up of cohorts	*	*	**--**	*	**--**
Overall (total number of asterisks)	8	7	5	8	5
**Case–Control Studies**					
**Items**	**Bhatti et al.** [11]	**Leung et al.** [15]			
Case definition	*	*			
Representativeness of cases	*	*			
Selection of controls	*	*			
Definition of controls	*	*			
Comparability	*	**			
Ascertainment of exposure	*	*			
The same method of ascertainment for cases and controls	*	*			
Nonresponse rate	*	*			
Overall (total number of asterisks)	8	9			

* This item was fulfilled (only comparability can have **); -- This item was not fulfilled.

**Table 3:** Sensitivity analyses were performed by leaving one study out and combining the remaining studies.

Removed Cohort	OR (95% CI)	*I* ^2^
Bhatti et al. [11]	1.07 (0.93, 1.23)	24.11%
Carter et al. [12]	1.09 (0.90, 1.32)	48.81%
Harris et al. [13]	1.18 (0.99, 1.41)	33.02%
Jørgensen et al. [14]	1.16 (1.00, 1.35)	42.41%
Leung et al. [15]	1.11 (0.92, 1.34)	55.19%
Poole et al. [NHS] [16]	1.15 (0.97, 1.37)	52.60%
Poole et al. [NHS II] [16]	1.12 (0.95, 1.33)	56.21%
Schwartzbaum et al. [17]	1.12 (0.95, 1.33)	56.32%

**Table 4:** Subgroup meta-analyses of the association between night shift work and ovarian cancer.

Factors		Number of Cohorts	OR (95% CI)	*I* ^2^
Study design	Case–control	2	1.36 (1.12, 1.66)	0.83%
	Cohort	6	1.04 (0.89, 1.23)	29.62%
Shift work assessment	Questionnaire	6	1.17 (0.96, 1.42)	43.89%
	Job-exposure matrix	2	1.00 (0.88, 1.14)	0.00%
Study quality	Moderate	2	0.81 (0.47, 1.40)	24.85%
	High	6	1.17 (1.00, 1.37)	52.01%
Outcome	Incidence	6	1.14 (0.95, 1.36)	42.96%
	Mortality	2	0.96 (0.49, 1.85)	77.72%

## Data Availability

No new data were created or analyzed in this study.

## Abbreviations

The following abbreviations are used in this manuscript:

IARC	International Agency for Research on Cancer
PRISMA	Preferred Reporting Items for Systematic Reviews and Meta-Analyses
PROSPERO	International Prospective Register of Systematic Reviews
OR	Odds Ratio
CI	Confidence interval
NOS	Newcastle–Ottawa Scale

## Appendix A

**Table A1:** Search terms.

Search (Shift work) AND (cancer) (“shift”[All Fields] OR “shifted”[All Fields] OR “shifting”[All Fields] OR “shiftings”[All Fields] OR “shifts”[All Fields]) AND (“work”[MeSH Terms] OR “work”[All Fields]) AND (“cancer s”[All Fields] OR “cancerated”[All Fields] OR “canceration”[All Fields] OR “cancerization”[All Fields] OR “cancerized”[All Fields] OR “cancerous”[All Fields] OR “neoplasms”[MeSH Terms] OR “neoplasms”[All Fields] OR “cancer”[All Fields] OR “cancers”[All Fields])
Translations Shift: “shift”[All Fields] OR “shifted”[All Fields] OR “shifting”[All Fields] OR “shiftings”[All Fields] OR “shifts”[All Fields] work: “work”[MeSH Terms] OR “work”[All Fields] cancer: “cancer’s”[All Fields] OR “cancerated”[All Fields] OR “canceration”[All Fields] OR “cancerization”[All Fields] OR “cancerized”[All Fields] OR “cancerous”[All Fields] OR “neoplasms”[MeSH Terms] OR “neoplasms”[All Fields] OR “cancer”[All Fields] OR “cancers”[All Fields]

## References

[1] Bray F., Laversanne M., Sung H., Ferlay J., Siegel R.L., Soerjomataram I., Jemal A. (2024). Global cancer statistics 2022: GLOBOCAN estimates of incidence and mortality worldwide for 36 cancers in 185 countries. CA Cancer J. Clin..

[2] Adjei N.N., Haas A.M., Sun C.C., Zhao H., Yeh P.G., Giordano S.H., Toumazis I., Meyer L.A. (2025). Cost of ovarian cancer by the phase of care in the United States. Am. J. Obstet. Gynecol..

[3] Teglia F., Collatuzzo G., Boffetta P. (2023). Occupational cancers among employed women: A narrative review. Cancers.

[4] IARC Working Group on the Evaluation of Carcinogenic Risks to Humans (2010). Painting, Firefighting, and Shiftwork.

[5] Moon J., Ikeda-Araki A., Mun Y. (2024). Night shift work and female breast cancer: A two-stage dose-response meta-analysis for the correct risk definition. BMC Public Health.

[6] Moon J., Holzhausen E.A., Mun Y. (2024). Risk of prostate cancer with increasing years of night shift work: A two-stage dose-response meta-analysis with duration of night shift work as exposure dose. Heliyon.

[7] Arafa A., Eshak E.S., Iso H., Muraki I., Tamakoshi A. (2021). Night Work, Rotating Shift Work, and the Risk of Cancer in Japanese Men and Women: The JACC Study. J. Epidemiol..

[8] Wang X., Ji A., Zhu Y., Liang Z., Wu J., Li S., Meng S., Zheng X., Xie L. (2015). A meta-analysis including dose-response relationship between night shift work and the risk of colorectal cancer. Oncotarget.

[9] Savvidis C., Koutsilieris M. (2012). Circadian rhythm disruption in cancer biology. Mol. Med..

[10] Schwarz C., Pedraza-Flechas A.M., Lope V., Pastor-Barriuso R., Pollan M., Perez-Gomez B. (2018). Gynaecological cancer and night shift work: A systematic review. Maturitas.

[11] Bhatti P., Cushing-Haugen K.L., Wicklund K.G., Doherty J.A., Rossing M.A. (2013). Nightshift work and risk of ovarian cancer. Occup. Environ. Med..

[12] Carter B.D., Diver W.R., Hildebrand J.S., Patel A.V., Gapstur S.M. (2014). Circadian disruption and fatal ovarian cancer. Am. J. Prev. Med..

[13] Harris M.A., MacLeod J., Kim J., Pahwa M., Tjepkema M., Peters P., Demers P.A. (2020). Use of a Canadian population-based surveillance cohort to test relationships between shift work and breast, ovarian, and prostate cancer. Ann. Work Expo. Health.

[14] Jørgensen J.T., Karlsen S., Stayner L., Hansen J., Andersen Z.J. (2017). Shift work and overall and cause-specific mortality in the Danish nurse cohort. Scand. J. Work Environ. Health.

[15] Leung L., Grundy A., Siemiatycki J., Arseneau J., Gilbert L., Gotlieb W.H., Provencher D.M., Aronson K.J., Koushik A. (2019). Shift work patterns, chronotype, and epithelial ovarian cancer risk. Cancer Epidemiol. Biomark. Prev..

[16] Poole E.M., Schernhammer E.S., Tworoger S.S. (2011). Rotating night shift work and risk of ovarian cancer. Cancer Epidemiol. Biomark. Prev..

[17] Schwartzbaum J., Ahlbom A., Feychting M. (2007). Cohort study of cancer risk among male and female shift workers. Scand. J. Work Environ. Health.

[18] Dun A., Zhao X., Jin X., Wei T., Gao X., Wang Y., Hou H. (2020). Association Between Night-Shift Work and Cancer Risk: Updated Systematic Review and Meta-Analysis. Front. Oncol..

[19] Moher D., Liberati A., Tetzlaff J., Altman D.G., PRISMA Group (2009). Preferred reporting items for systematic reviews and meta-analyses: The PRISMA statement. PLoS Med..

[20] Wells G., Shea B., O’Connell D., Peterson J., Welch V., Losos M., Tugwell P. The Newcastle Ottawa Scale (NOS) for Assessing the Quality of Nonrandomized Studies in Meta-Analyses. https://www.ohri.ca/programs/clinical_epidemiology/oxford.asp.

[21] DerSimonian R., Laird N. (1986). Meta-analysis in clinical trials. Control. Clin. Trials.

[22] Higgins J., Thompson S., Deeks J., Altman D. (2003). Measuring inconsistency in meta-analyses. BMJ.

[23] Egger M., Davey Smith G., Schneider M., Minder C. (1997). Bias in meta-analysis detected by a simple, graphical test. BMJ.

[24] Viechtbauer W. (2010). Conducting meta-analyses in R with the metafor package. J. Stat. Softw..

[25] Grimes D.A., Schulz K.F. (2002). Bias and causal associations in observational research. Lancet.

[26] Barry D. (1996). Differential recall bias and spurious associations in case/control studies. Stat. Med..

[27] Lizama N., Heyworth J., Thomson A., Slevin T., Fritschi L. (2017). Self-reported shift work, recall bias, and belief about disease causation in a case-control study of breast cancer. Cancer Epidemiol..

[28] Zhao L., Tang Y., Yang J., Lin F., Liu X., Zhang Y., Chen J. (2023). Integrative analysis of circadian clock with prognostic and immunological biomarker identification in ovarian cancer. Front. Mol. Biosci..

[29] Xu Y.X., Shen Y.T., Li J., Ding W.Q., Wan Y.H., Su P.Y., Tao F.B., Sun Y. (2024). Real-ambient bedroom light at night increases systemic inflammation and disrupts circadian rhythm of inflammatory markers. Ecotoxicol. Environ. Saf..

[30] Depner C.M., Stothard E.R., Wright K.P. (2014). Metabolic consequences of sleep and circadian disorders. Curr. Diab. Rep..

[31] Liu H., Liu Y., Hai R., Liao W., Luo X. (2022). The role of circadian clocks in cancer: Mechanisms and clinical implications. Genes Dis..

[32] Tsai R.J., Luckhaupt S.E., Sweeney M.H., Calvert G.M. (2014). Shift work and cancer screening: Do females who work alternative shifts undergo recommended cancer screening?. Am. J. Ind. Med..

[33] Ferguson J.M., Bradshaw P.T., Eisen E.A., Rehkopf D., Cullen M.R., Costello S. (2023). Distribution of working hour characteristics by race, age, gender, and shift schedule among U.S. manufacturing workers. Chronobiol. Int..

